# Characterization and modeling of additively manufactured Ti-6Al-4V alloy with modified surfaces for medical applications

**DOI:** 10.3389/fbioe.2025.1526873

**Published:** 2025-04-07

**Authors:** Hüray Ilayda Kök, Tonya Andreeva, Sebastian Stammkötter, Cindy Reinholdt, Osman Akbas, Anne Jahn, Florian Gamon, Sandra Fuest, Mirko Teschke, Miriam Schäfer, Michael Müller, Alexander Koch, Ole Jung, Mike Barbeck, Andreas Greuling, Ralf Smeets, Jörg Hermsdorf, Rumen Krastev, Philipp Junker, Meike Stiesch, Frank Walther

**Affiliations:** ^1^ Leibniz University Hannover, Institute of Continuum Mechanics, Hannover, Germany; ^2^ Reutlingen University, Faculty of Life Sciences, Reutlingen, Germany; ^3^ TU Dortmund University, Chair of Materials Test Engineering (WPT), Dortmund, Germany; ^4^ Clinic and Polyclinic for Dermatology and Venerology, University Medical Center Rostock (UMR), Rostock, Germany; ^5^ Department of Prosthetic Dentistry and Biomedical Materials Science, Hannover Medical School, Hannover, Germany; ^6^ Additive Manufacturing Department, Laser Zentrum Hannover e.V., Hannover, Germany; ^7^ Department of Oral and Maxillofacial Surgery, University Hospital Hamburg-Eppendorf, Hamburg, Germany; ^8^ Department of Oral and Maxillofacial Surgery, Division of Regenerative Orofacial Medicine, University Hospital Hamburg-Eppendorf, Hamburg, Germany

**Keywords:** additive manufacturing, Ti-6Al-4V alloy, osseointegration, dental implants, surface modification, fatigue behavior, finite element method

## Abstract

In the field of biomedical implants, additively manufactured titanium alloys, particularly Ti-6Al-4V, hold significant potential due to their biocompatibility and mechanical properties. This study focuses on the characterization and modeling of additively manufactured Ti-6Al-4V alloy for dental and maxillofacial implants, emphasizing fatigue behavior, surface modification, and their combined effects on cyto- and osseocompatibility. Experimental methods, including tensile, compression, and fatigue testing, were applied alongside *in silico* simulations to assess the long-term mechanical performance of the material. Surface properties were further modified through sandblasting and coating techniques to enhance cell adhesion and proliferation. By using in-vitro methods, the cytocompatibility of the coatings and materials was examined followed by in-vivo tests to determine osseocompatibility. Results demonstrated that appropriate surface roughness and modifications are essential in optimizing osseointegration, while the layer-by-layer additive manufacturing process influenced the fatigue life and stability. These findings contribute to the development of patient-specific implants, optimizing both mechanical integrity and biological integration for enhanced clinical outcomes. This work summarizes the investigations on additively manufactured Ti-6Al-4V alloy of the research unit 5250 “Mechanism-based characterization and modeling of permanent and bioresorbable implants with tailored functionality based on innovative *in vivo*, *in vitro* and *in silico* methods” funded by the Germany Research Foundation (DFG).

## 1 Introduction

In the field of dentistry and oral and maxillofacial surgery, the implantation of permanent or bioabsorbable implants is the preferred procedure for replacing lost hard tissue due to trauma, tumors, or malformations (Glenske et al., 2018). The success of these procedures relies on a range of factors, beyond the implant itself, including osseointegration and mechanical performance. Biocompatibility of the implant material, which is essential for preventing the body’s immune system from rejecting the implant. Furthermore, the (nano) surface characteristics and the structural topography of the implant surface, in consideration of its overall design, are of critical importance for the realization of osseointegration and the assurance of long-term stability (Parithimarkalaignan and Padmanabhan, 2013). A variety of surface modification techniques have been explored to enhance the performance of titanium alloys, including methods such as chemical etching, anodization, and advanced coatings. Notable examples include the works by Xu et al. (2021) and Xu et al. (2022), which provide innovative approaches to surface engineering of titanium alloys for biomedical applications.

Titanium alloys, such as Ti-6Al-4V, are frequently used in biomedical applications due to their exceptional mechanical properties and biocompatibility (Kotzem et al., 2023; Kotzem et al., 2021; Sidambe, 2014). However, in response to the growing complexity of patient-specific requirements in surgical procedures, additive manufacturing (AM) has emerged as a promising manufacturing process. AM enables the manufacturing of complex geometries, including lattice structures, which can be employed to modify material properties, particularly stiffness (Koju et al., 2022), and to mitigate the risk of stress shielding (Noyama et al., 2012; Niinomi and Nakai, 2011).

To mitigate this effect, lattice structures have been demonstrated to reduce the stiffness of the implant while simultaneously enhancing nutrient transport and vascularization, which are essential for osseointegration and tissue regeneration (Deering and Grandfield, 2021; Pei et al., 2022; Wang et al., 2022). Nevertheless, any reduction in stiffness and modifications to the implant’s shape must be achieved without compromising the functionality. It is therefore essential to investigate the fatigue characteristics of additively manufactured specimens to determine the optimal design (Wegner et al., 2019). The powder bed fusion laser-based process of metal (PBF-LB/M) process inherent to AM has the potential to introduce microstructural anisotropies or residual stresses, which may ultimately impact the fatigue life of the material (Chen et al., 2022; Yasin et al., 2024; Beretta and Romano, 2017; Bologna et al., 2024; Centola et al., 2023). Therefore, a comprehensive evaluation of the fatigue characteristics is essential to guarantee the long-term functionality of the material under cyclic loading conditions.

Following surgical implantations, an undisturbed healing phase is essential for the successful integration of the implant with the surrounding bone matrix growth and tissues. Implant loading conditions must be carefully controlled to prevent overstressing the bone during the early healing period, which could result in implant failure (Staedt et al., 2020). In addition to experimental studies, numerical simulations are crucial for assessing the long-term fatigue behavior of implants by replicating patient-specific loading conditions. These simulations guide focused experiments and optimize the implant design before clinical application.

The surface characteristics of the implant, including micro- and nano-roughness, structural morphology, and chemical composition, are also of critical importance in enhancing osseointegration and promoting bone formation.

Regardless of the material employed, surface topography is of primary importance for successful tissue regeneration and healing (Zhang et al., 2023). Research has demonstrated that the proliferation, differentiation, morphology, and matrix production of osteoblast cells can be markedly enhanced by optimizing surface roughness profiles (Kunzler et al., 2007; Karageoriou and Kaplan, 2005). In particular, surface roughness *R*_a_ within the range of 1–2 μm has been demonstrated to be effective in promoting osseointegration, as it closely resembles the natural topography of bone. Moreover, the integration of macro-, micro-, and nanoscale structures can enhance the directionality and temporal control of tissue regeneration at the cellular level (Yadroitsev et al., 2014).

The objective of this paper is to examine the interrelationship between the fatigue characteristics of additively manufactured Ti-6Al-4V alloy, including both experimental and simulation-based studies, surface characteristics, and their collective impact on the cyto- and osseocompatibility. These studies were performed in a team of researchers united in the research unit 5250 “Mechanism-based characterization and modeling of permanent and bioresorbable implants with tailored functionality based on innovative *in vivo*, *in vitro* and *in silico* methods” funded by the Germany Research Foundation (DFG).

## 2 Methods and materials

This section presents methods for characterizing the properties of Ti-6Al-4V specimens to promote osseointegration. The first section addresses the mechanical properties of additively manufactured Ti-6Al-4V specimens to ensure long-term stability. The second part focuses on the surface properties of various specimens, including as-built specimens with varying roughness levels controlled by additive manufacturing parameters. Additionally, machined specimens with smooth surfaces were prepared by cutting titanium rods, and some of these machined specimens were further modified through sandblasting to create rougher surfaces. Different coating strategies were applied to additively manufactured, machined, and sandblasted specimens to further enhance surface properties and promote osseointegration. This comprehensive approach allowed for the investigation of a wide range of surface roughnesses, textures, and coating effects. The section also examines the interaction between mechanical and biological properties, followed by methods used for biological characterization.

### 2.1 Manufacturing

#### 2.1.1 Additive manufacturing

The Ti-6Al-4V specimens were additively manufactured *via* powder bed fusion laser-based process (PBF-LB/M) using the industrial machine Lasertec 12 SLM by DMG MORI at Laser Zentrum Hannover e.V. (LZH, Hannover, Germany). The machine is equipped with a 400 W fiber laser emitting at 1,070 nm in continuous wave mode, featuring a minimum spot diameter of 35 µm. The used powder specification is Ti-6Al-4V Grade 23 (ECKART TLS GmbH, Bitterfeld-Wolfen, Germany) and exhibits a predominantly spherical morphology, with particle sizes ranging from 20.0 to 53.0 µm.

**FIGURE 1:**
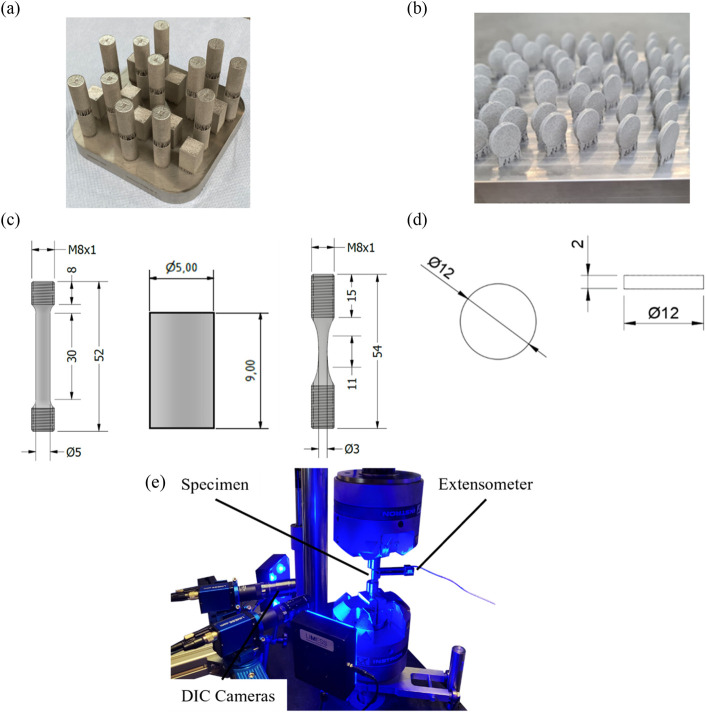
Additive manufacturing build job design of Ti-6Al-4V alloy: **(a)** Specimens for tensile and compression testing; **(b)** Discs for cell testing; dimensions of Ti-6Al-4V specimens for **(c)** tensile, compression and fatigue testing; **(d)** machined discs; **(e)** testing setup for investigations of Ti-6Al-4V.

Different shaped specimens were manufactured for the experiments (Figure 1).

a) for the mechanical characterization cylinders for tensile and compression testing
b) for biological experiments, discs (with 12 mm diameter and 2 mm thickness) with three different surface roughnesses (*S*_a_).

The process parameters are listed in Table 1. All specimens were manufactured in an upright position with a bidirectional scan strategy.

**TABLE 1:** PBF-LB/M manufacturing parameters of Ti-6Al-4V alloy

Part	Scan path	Parameter	
		Laser power [W]	Scan speed [mm/s]
Specimen for tensile/compression tests (Figure 1A)	Contour/Hatch	200	1,000
Discs for cell tests (Figure 1B)			
Roughness 1: AMTi12 (*S*_a_ 12.52 µm)	Contour	200	1,000
	Hatch	175	1,050
Roughness 2: AMTi15 (*S*_a_ 14.64 µm)	Contour	120	1,200
	Hatch	175	1,050
Roughness 3: AMTi17 (*S*_a_ 17.40 µm)	Contour	40	287
	Hatch	175	1,050

The final dimensions of the specimens can be extracted from Figures 1c, d.

After the manufacturing process and ultrasonic cleaning, additional heat treatment was conducted to reduce manufacturing-related residual stresses and to guarantee a homogeneous and stable microstructure based on ISO 20160. The heat treatment was performed in a vacuum furnace at 1,050°C for 4 h with conventional furnace cooling afterward (Chunze et al., 2021). The surface roughness levels were quantified using a laser-scanning confocal microscope (Keyence VK-X1,000), measuring the arithmetic mean roughness (Sa) over an area of 1.3 mm^2^ on the side surfaces of the samples. Preliminary investigations showed that the ratio between the roughness parameters Sz (maximum height) and Sa (arithmetic mean roughness) remained consistent across all samples. Based on this observation, Sa was chosen as the representative roughness parameter, as it effectively describes the average surface structure while facilitating consistent comparison between samples. Generally, the surface roughness of additively manufactured parts differs based on a variety of factors. Based on the process capability and the target to examine different surfaces, but using the same equipment, material and design the roughness levels were determined by only varying the process parameters. The three roughness levels represented a progressive increase of 12.52 µm (AMTi12), 14.64 µm (AMTi15) and 17.40 µm (AMTi17) to comprehensively investigate the relationship between surface roughness and biological properties.

#### 2.1.2 Machining and sandblasting

The specimens were cut from a Ø12 mm Ti-6Al-4V rod (L. Klein SA, Biel, Switzerland) using a linear precision saw (Brillant 220, ATM Qness GmbH, Mammelzen, Germany). A diamond cutting disc (Buehler, ITW Test & Measurement GmbH, Leinfelden-Echterdingen, Germany) was used and the specimen thickness was 2 mm. The samples were deburred afterward in a rotary tumbler (Model 45C, Lortone Inc., Meridian, Idaho, United States), resulting in the geometry shown in Figure 1d).

For the sandblasted group, one side of each specimen was sandblasted using a sandblasting machine from a previous study (Finger et al., 2020), following a meander-shaped sandblasting path with a 0.3 mm offset and a travel speed of 1 mm/s, resulting in a sandblasting time of approx. 8 min per specimen. Sandblasting was carried out at an angle of 90° and a distance of 15 mm, with a pressure of 2 bar. After sandblasting, the surface roughness was measured with an optical profilometer (MicroProf 100; FRT GmbH, Bergisch-Gladbach, Germany), and the topography was analyzed using scanning electron microscopy (SEM) (EVO MA10; Carl Zeiss AG, Oberkochen, Germany). Imaging was conducted at × 500 magnification over an area of 450 × 600 μm^2^, enabling detailed inspection of the surface topography and assessment of microstructural changes.

### 2.2 Testing setup for quasi-static and fatigue experiments

To characterize the solid Ti-6Al-4V material, quasi-static compression and tensile tests, as well as fatigue loading tests were performed using Instron 8801 (Instron GmbH, Darmstadt, Germany) with load cell of ±100 kN. The specimen geometries used for the tensile, fatigue, and compression tests are illustrated in Figure 1c) and the testing setup in Figure 1e). The strains were measured using an extensometer (Instron GmbH, Darmstadt, Germany) with an initial gauge length of l_0_ = 12.5 mm with ± 1 mm travel as well as digital image correlation. All tests were performed according to DIN 6892–1 and DIN 50106. Specific quasi-static properties like Young’s modulus, fracture strain, and ultimate strength were determined. Fatigue tests were carried out at a frequency of *f* = 10 Hz under stress control.

This subsection presents a novel and effective approach for modeling fatigue, addressing the limitations of conventional cycle-by-cycle simulations, which frequently rely on extensive processing times. It presents an overview of the evolution equations that form the foundation of the material model. Thereby, it establishes the theoretical framework for fatigue life assessment and long-term stability of the additively manufactured specimens. The objective of this methodology is to enhance the accuracy of fatigue predictions while significantly reducing computational and experimental costs.

#### 2.2.1 Extended hamilton principle

The damage model for fatigue is founded upon the extended Hamilton principle (Junker and Balzani, 2021). In the isothermal case, the extended Hamilton functional is given by

H≔G+D+C. (1)

It can be defined as the summation of functionals, which include the Gibbs energy G , dissipation energy D and additional constraints that can be incorporated into the constraint functional C .

The Gibbs energy

$$G\left[u,\nu \right]=\int_{\Omega }\psi \left(\varepsilon ,\nu \right)\;dV-\int_{\Omega }b^{*}\cdot u\;dV\int_{\partial \Omega }t^{*}\cdot u\;dA$$ (2)

can be defined as the difference between the Helmholtz free energy ψ stored in the volume of the body Ω and the work of the external loads, given by body $b^{*}$ and traction forces $t^{*}$ at the Neumann boundary ∂Ω .

The dissipation functional is defined as

$$D\left[\nu \right]=\int_{\Omega }p^{\text{diss},*}\cdot \nu \;dV\text{ with }p^{diss,*}=\frac{\partial \Delta^{\text{diss}}}{\partial \nu }.$$ (3)

The remaining term for the current material model accounts of volume conservation, which is included as a constraint. Therefore, the constraint functional denotes

$$C\left[\nu \right]=\int_{\Omega }p^{c,*}\cdot \nu \;dV\text{ with}{\;p}^{c,*}=\frac{\partial c}{\partial \nu }.$$ (4)

The stationarity of this principle results in field equations for the displacement u as well as all state variables, including internal variables ν (Junker and Wick, 2023). As a result, it includes the evolution of microstructural material behavior with regard to the internal variable ν , represented by evaluation of the stationarity condition

$$H\left[u,\nu \right]\to \underset{u,\nu }{stat}\Leftrightarrow \delta H=0\;\forall \;\delta u,\delta \nu .$$ (5)

In this context of a quasi-static process, dynamic effects are excluded. The stationarity demands due to the independence of the respective test functions δu and δν

$$\left\{\begin{array}{l} \delta_{u}H=0\;\forall \;\delta u\\ \delta_{\nu }H=0\;\forall \;\delta \nu . \end{array}\right.$$ (6)

Computing the stationarity and employing integration by parts, we obtain the generalized evolution equation for the internal variables as

$$\left\{\begin{array}{l} \frac{\partial {∆}^{\text{diss}}}{\partial \dot{\nu }}+\frac{\partial \psi }{\partial \nu }-\nabla \cdot \frac{\partial \psi }{\partial \nabla \nu }+\frac{\partial c}{\partial \dot{\nu }}∋0\;\forall x\in \Omega \\ \;n\cdot \frac{\partial \psi }{\partial \nabla \nu }=0\;\forall x\in \partial \Omega . \end{array}\right.$$ (7)

It can be evaluated as soon as the dissipation function ${∆}^{\text{diss}}$ and the free energy ψ density are specified for a specific material.

#### 2.2.2 Transformation of time *t* to cycles *N*

Our aim is the modeling of fatigue. Thus, the generalized evolution equation could be used to model the evolution of microstructure in the course of time. However, this approach would consume a remarkable amount of computational effort which practically prevents the simulation of high-cycle fatigue problems with millions of cycles. Hence, to reduce computational expenses, we implement a coordinate transformation from time *t* → *N*.

$$N=\omega t\;\Rightarrow t=\frac{N}{\omega }\;\Rightarrow dt=\frac{dN}{\omega }$$ (8)

For any time, derivatives $\dot{∎}$ , it transforms as

$$\dot{∎}=\frac{d}{dt}∎=\omega \frac{d}{dN}∎=:\omega \tilde{∎}.$$ (9)

Then, the stationarity condition for the internal variables from Equation 5 is reformulated

$$\frac{1}{\omega }\frac{\partial {∆}^{\text{diss}}}{\partial \tilde{\nu }}+\frac{\partial \psi }{\partial \nu }-\nabla \cdot \frac{\partial \psi }{\partial \nabla \nu }+\frac{1}{\omega }\frac{\partial c}{\partial \tilde{\nu }}∋0\;\forall x\in \Omega .$$ (10)

#### 2.2.3 Specification of the material model

For the specific model for fatigue processes, the following internal variables are introduced $\nu ≔\{d,\varepsilon_{p}\left.,h\right\}$ with d as the damage variable, $\varepsilon_{p}$ as the plastic strain and h the hardening parameter.

As part of the Gibbs energy, the Helmholtz free energy ψ is separated into three parts: mechanical, regularization, and hardening contributions, i.e.,

$$\psi \left(\varepsilon ,\nu \right)=\frac{1}{2}\left(\varepsilon -\varepsilon_{p}\right):f\left(d\right)C:\left(\varepsilon -\varepsilon_{p}\right)+\frac{1}{2}\beta \;{\left\|\nabla f\left(d\right)\right\|}^{2}+\frac{1}{2}kh^{2},$$ (11)

which include the monotonically decreasing damage function f

$$f\left(d\right)=e^{-d}.$$ (12)

The dissipation function is a homogeneous function of first and second order, resulting in a differential inequality for the damage function and a differential algebraic system of equations for the plastic strains. The dissipation function is defined as follows:

$$\Delta^{\text{diss}}=r_{p}\omega \left\|{\tilde{\varepsilon }}_{p}\right\|+\frac{1}{2}\eta_{p}\omega^{2}{\left\|{\tilde{\varepsilon }}_{p}\right\|}^{2}+r_{d}\omega \tilde{d}+\frac{1}{2}\eta_{d}\omega^{2}{\left|\tilde{d}\right|}^{2}$$ (13)

with r as the dissipation parameter respectively for damage and plasticity and η the viscosity parameter.

Finally, the stationarity conditions from Equation 5 and Equation 7 constitutes as

$$\left\{\begin{array}{l} r_{p}\partial {\tilde{\varepsilon }}_{p}+\eta_{p}\omega {\tilde{\varepsilon }}_{p}-\sigma +\gamma_{1}I{\tilde{\varepsilon }}_{p}-\gamma_{2}\partial {\tilde{\varepsilon }}_{p}∋0\\ k\tilde{h}+\gamma_{2}=0\\ r_{d}\partial \tilde{d}+\eta_{d}\omega \tilde{d}-f\;\psi_{0}-\beta f\;\nabla^{2}f∋0 \end{array}\right.$$ (14)

where $\delta_{u}H=0$ is the balance of linear momentum and the evolution equation for the damage function in its strong form forms to

$$\eta_{d}\omega \tilde{d}-f\;\psi_{0}-\beta f\;\nabla^{2}f\leq r_{d}$$ (15)

followed from $\delta_{d}H=0$ . The plasticity material law is given by $\delta_{\varepsilon_{p}}H=0$ . The coupled system of equations is solved using the Neighbored Element Method (NEM) (Blaszczyk et al., 2022; Vogel and Junker, 2020).

#### 2.2.4 Simulation parameter

**FIGURE 2:**
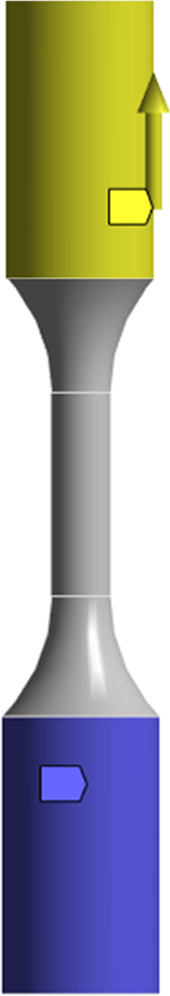
Boundary conditions for the hourglass specimen: fixed boundary conditions at the bottom (blue) and displacement boundary condition at the top (yellow).

The *in silico* fatigue assessment was conducted to improve prediction accuracy compared to traditional methods. The assessment involved a systematic evaluation of the relationship between stress amplitude and fracture cycle count. Stress amplitudes ranging from 350 MPa to 650 MPa were applied to construct S-N (Wöhler) curves systematically. For constructing the S-N curve, individual simulations were conducted for each point on the curve, with different load amplitudes applied at each step. The hourglass shaped specimen (compare Figure 1c) is used for the fatigue simulation. The boundary conditions are applied as shown in Figure 2. These amplitudes are detailed in Supplementary Table S1. The material parameters for Young’s modulus, Poisson’s rate, and dissipation are provided in Table 2.

**TABLE 2:** Material properties for the simulation.

Quantity	Symbol	Value
Young’s modulus-tensile	E	110 GPa
Poisson’s ratio	v	0.32
Regularization parameter	β	0.1 N
Fatigue rate parameter	$\eta_{d}$	0.2 MPa
Plasticity rate parameter	$\eta_{p}$	100 GPa
Damage dissipation parameter	$r_{d}$	6 Pa
Plasticity dissipation parameter	$r_{p}$	100 Pa
Hardening parameter	h	80 Pa

The failure cycle, $N_{f}$ , was defined as the cycle where the reaction force met the criterion $|F_{t}|<0.1\cdot F_{t}$ , indicating that the material had reached failure.

### 2.3 Surface modification

#### 2.3.1 Sand-blasting

In Figure 1d discs are used to modify the surface by sand-blasting. One side of each disc was sandblasted using a modified 3D printer with a sandblasting nozzle, following a meander-shaped path with a 0.3 mm offset at 1 mm/s. Sandblasting was performed at 90° and 15 mm distance with pressures of 2 bar and 6 bar, creating two distinct roughness levels. Average surface roughness was measured with an optical profilometer, and topography was assessed *via* scanning electron microscopy (SEM).

#### 2.3.2 Coating

A polyelectrolyte (PE) multilayer (PEM) coating system was applied for chemical modification of the surface. It was assembled by application of a layer-by-layer technology consisting of alternating deposition of polyanion polyacrylic acid, PAA (100 kDa, 35%wt, Sigma Aldrich, Steinheim, Germany) and polycation poly (allylamine hydrochloride), PAH (120–200 kDa, Life Technologies GmbH, Darmstadt, Germany) adopting the protocol described in (Bradford, 1976). The overall coating composition was (PAA/PAH)_5_ and the number of five bilayers was selected to ensure that the PEM coating is homogeneous and independent of the type and properties of the substrate (Smith et al., 2011).

For physicochemical characterization, a PEM coating was applied on Si-wafers (10 × 10 mm^2^, CrysTec GmbH, Berlin, Germany) pre-cleaned by successive ultrasonication in acetone and 2-propanol. For cell culture experiments, the same coating was built in sterile conditions on machined Ti-6Al-4V discs (MTi), sand-blasted Ti-6Al-4V discs (SBTi), and additively manufactured Ti-6Al-4V discs (AMTi) with three different levels (Table 1) of AMTi12, AMTi15 and AMTi17, all cleaned successively in hot (60°C) solution of 10 mM SDS, acetone and 2-propanol.

The (PAA/PAH)_5_ coating was applied here for chemical modification of the surface. It was assembled by application of the layer-by-layer technology consisting of alternating deposition of polyanion (PAA) and polycation (PAH) adopting the protocol described in (Hartmann et al., 2013). The number of five bilayers was chosen to ensure that the PEM coating is homogeneous and independent of the type and properties of the substrate (Ladam et al., 1999).

The thickness of the coating was measured by ellipsometry (SENTECH Instruments GmbH, Berlin, Germany) in a dry state. The hydrophilicity was analyzed by static water contact angle measurements (DataPhysics Instruments GmbH, Filderstadt, Germany) by applying the Young–Laplace fitting procedure. Atomic force microscopy (AFM) (Oxford Instruments plc, Abingdon, United Kingdom) was utilized for assessing the average surface roughness. The surface density of human serum albumin (HSA) on the coating was analyzed by following the Bradford assay (Bradford, 1976).

The adhesion strength of PEM coatings on MTi and AMTi15 materials was evaluated using an automatic pull-off adhesion tester (PosiTest AT-A, DeFelsko) in accordance with ASTM F1147-05 standards. This method measures the tensile force required to cause adhesive or cohesive failure between two adhesive pull-off cylinders: one AMTi cylinder coated with the PEM coating and one uncoated control cylinder. Both cylinders were bonded using a high-adhesive epoxy glue, with uncoated cylinders serving as controls.

### 2.4 *In vitro* cytocompatibility

Cells viability and proliferation on the uncoated and PEM-coated Ti-6Al-4V specimen (machined, sand-blasted and additively manufactured) with different roughness levels were investigated with 4 cell types - L929 fibroblasts (cell lines service GmbH, Eppelheim, Germany, Cat. No.: 400260–817), MC3T3 preosteoblasts (American Type Culture Collection, Virginia, United States, Cat. No.: CRL-2593), human dental pulp stem cells (hDPSCs), and human osteoblasts (NHOst) (Lonza Group AG, Basel, Switzerland, Cat No.: CC-2538) based on previously described protocols (Alkildani et al., 2021; Jung et al., 2020). Extracts from each specimen and control group were incubated for 24 h in the appropriate cell type specific medium under cell culture conditions (37°C, 5% CO_2_ and 95% humidity). The cells were then incubated with the extracts for a further 24 h and analyzed immediately. The following established methods were employed in order to ascertain the cytocompatibility of these cell types: Lactate dehydrogenase (LDH) activity assays were performed to assess the adhesion and cell growth on PEM-coated *versus* uncoated titanium and on smoother *versus* rougher titanium. LDH assay was carried out following the protocol described in detail in (Longhitano et al., 2018). 2,3-bis-(2-methoxy-4-nitro-5-sulfophenyl)-2H-tetrazolium-5-carboxanilide (XTT) reduction assay was performed according to the protocol provided by the manufacturer (Thermo Fisher Scientific, Waltham, Massachusetts, United States).

XTT assay and LDH assay are commonly used methods to assess cytotoxicity and cell viability in biomedical research. The XTT assay measures the metabolic activity of cells by quantifying the reduction of a colorimetric dye, providing an indirect measurement of cell proliferation and viability. In contrast, the LDH assay detects the release of lactate dehydrogenase (LDH), an enzyme presents in the cytoplasm, upon cell membrane damage or lysis, indicating cellular toxicity or damage. Both assays offer valuable insights into the health and functionality of cells when exposed to various treatments or materials, aiding in the evaluation of biocompatibility and potential cytotoxic effects.

The cultivation and differentiation of hDPSCs is considered a stem cell model with potential for osteogenic, chondrogenic, and adipogenic differentiation (Al-Sanabani et al., 2013) (Supplementary Figure S1).

### 2.5 *In vivo* biocompatibility

The subcutaneous implantation study was performed *via* an established methodology (Barbeck et al., 2023; Alkildani et al., 2022; Barbeck et al., 2024). Briefly, the additively manufactured Ti-6Al-4V with three different roughnesses were inserted in a subcutaneous tissue pocket using 30 female 5-week-old Wistar rats (n = 5 animals for 10 and 30 days post implantation) after the approval of the Local Ethical Committee of the Faculty of Medicine at the University of Niš, Serbia, based on the approval of the Veterinary Directorate of the Ministry of Agriculture, Forestry and Water Management of the Republic of Serbia (approval number 323–07-01762/2019–05/9; date of approval: 01 March 2019). Thereby, experimental animals were housed using standard conditions with regular mouse pellets, access to water *ad libitum*, and an artificial light-dark cycle of 12 h each. After intraperitoneal anesthesia [10 mL of ketamine (50 mg/mL) with 1.6 mL of 2% xylazine] the subcutaneous implantation included the insertion of the Ti-6Al-4V into a preformed subcutaneous pocket within the rostral subscapular region under sterile conditions followed by wound closure with Prolene 6.0 (Ethicon, Somerville, NJ, United States). After the respective study time points, the experimental animals were sacrificed by an overdose of ketamine, xylazine, and the implanted biomaterials were extracted together with the peri-implant tissue and fixated in 4% formalin for 24 h. The explants were embedded in paraffin, cut with a rotation microtome (Leica, Wetzlar, Germany) in 3–5 μm thick slices, and stained with hematoxylin and eosin (H&E). Afterward, the tissue sections were analyzed histologically with a conventional diagnostic microscope (Eclipse 80i, Nikon, Tokyo, Japan) in combination with a digital camera (DS-Fi1, Nikon, Tokyo, Japan) with a digital sight control unit (Nikon, Tokyo, Japan).

## 3 Results and discussion

### 3.1 Mechanical behavior

**FIGURE 3:**
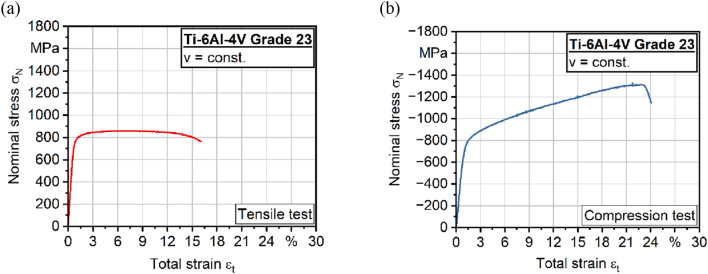
**(a)** Quasi-static tensile and **(b)** compression tests of Ti-6Al-4V alloy.

The heat-treated and therefore stress-relieved condition of the specimen shown in Figure 1c) was initially characterized in the case of quasi-static compression and tensile behavior to evaluate the material response in different loading conditions. Figure 3 presents the tensile test results which align with the findings of previous studies (Longhitano et al., 2018; Nes et al., 2022). Focusing on the determined properties, Young’s modulus of E ≈ 110,000 MPa was reached and is comparable to other studies (Nes et al., 2022; Kasperovich and Hausmann, 2015; Tao et al., 2018). Further parameters like fracture strain (FS) were determined with FS ≈ 14.8% while the ultimate tensile strength (UTS) was estimated with UTS ≈ 870 MPa. When comparing the tensile and compression behavior of the same material condition, an increased ultimate compression strength to UCS ≈ −1,250 MPa can be detected. As a result of the compression tests, the elongation at break (FS) also increases up to FS ≈ 24% which is comparable to the literature (Lee et al., 2022).

**FIGURE 4:**
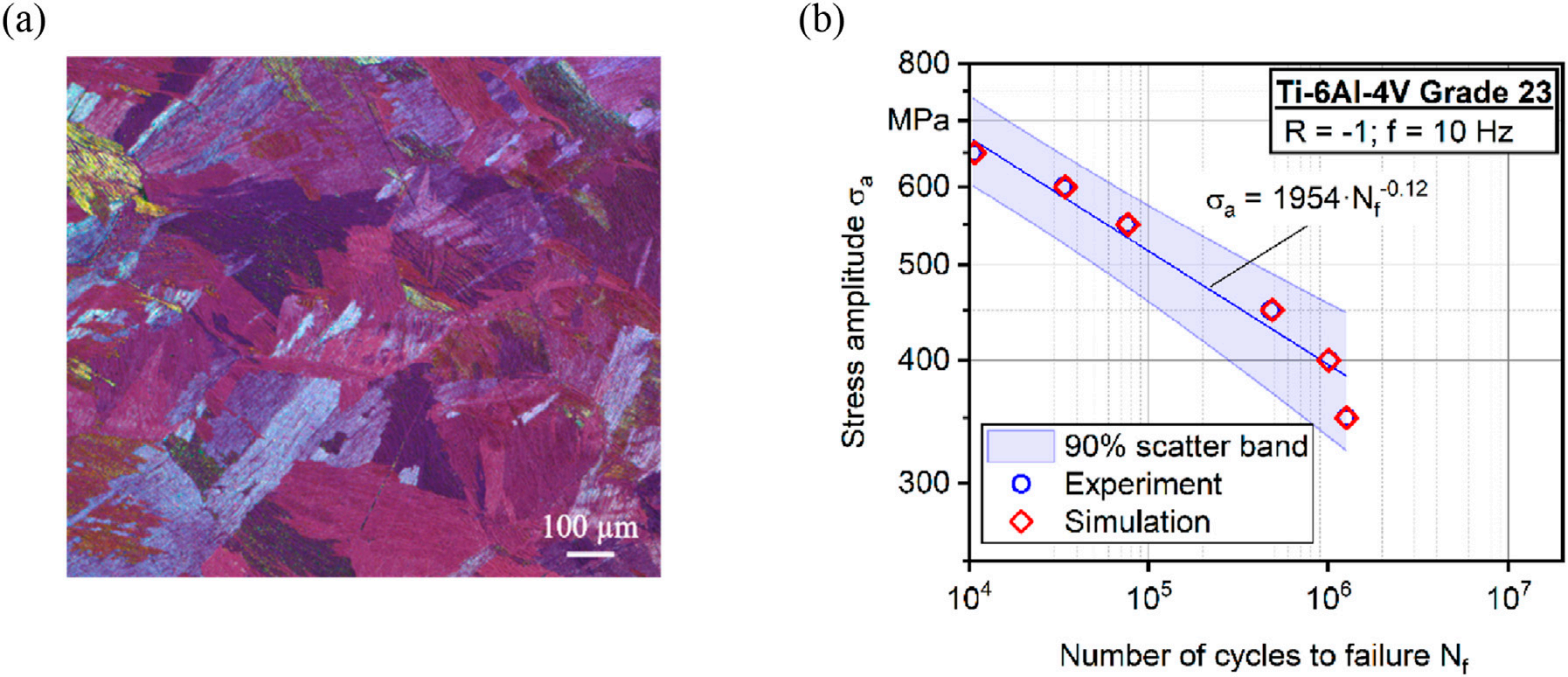
**(a)** Microstructure after heat treatment and **(b)** experimental S-N (Woehler) curve of Ti-6Al-4V alloy.

In addition to quasi-static tensile and compression tests, microstructural analyses were performed to initially characterize the phase distribution after heat treatment. Figure 4a) shows the microstructure of Ti-6Al-4V Grade 23 (ELI) with a homogenous structure. It also indicates an α+β microstructure that can be reached after super-transus heat treatment (Okabe and Hero, 1995).

To evaluate the performance under cyclic loading, additional fatigue tests were carried out. Figure 4b) shows the results of constant amplitude tests (CAT) in high cycle fatigue (HCF) regime. The samples exhibit a strong correlation with the Basquin fit, effectively capturing the fatigue behavior of Ti-6Al-4V. This correlation enables an accurate description of the material’s properties under cyclic loading.

Damage models and models for tissue and implant interface are essential for the appropriate design of patient-specific implants. The fatigue results are used for experimental verification of numerical simulations.

### 3.2 Numerical behavior

The *in silico* fatigue assessment yielded a significant improvement in prediction accuracy compared to traditional methods. The relationship between stress amplitude and number of cycles to failure was systematically evaluated. The S-N curve shown in Figure 4b) illustrates the fatigue properties as a function of stress amplitude.

At a stress amplitude of 350 MPa, the material withstood approx. $N_{f}=1,261,680$ cycles before failure in the simulation, while increasing the stress to 400 MPa reduced the number of cycles to $N_{f}=1,004,055$ . Further increases in the stress amplitudes resulted in a sharp decline in fatigue lifetimes, with $N_{f}=485,810$ cycles at 450 MPa and $N_{f}=34,536$ cycles at 600 MPa. These values are consistent with the general trend observed in the experimental studies above.

### 3.3 Surface roughness

#### 3.3.1 Ti-6Al-4V specimens

Surface characteristics of the implanted materials such as chemical composition, surface energy (hydrophilicity), and roughness should be considered to ensure successful and long-term osseointegration (Boyan et al., 2017). Among them, the micro- (<100 μm), submicron (<1 μm), and nanoscale roughness (<100 nm) were shown to have the primary role (Boyan et al., 2003).

Five Ti-6Al-4V specimens with various roughness levels, each with the geometry shown in Figure 1d), were produced and studied: smooth MTi-discs with average *S*_a_ of 0.50 µm, SBTi-discs with *S*_a_ of 2.06 µm, and three types of AMTi-discs with *S*_a_ of 12.52 µm, 14.64 µm and 17.40 µm **(** Table 1 **)**. Si-wafers were used in some cases as a model substrate.

**FIGURE 5:**
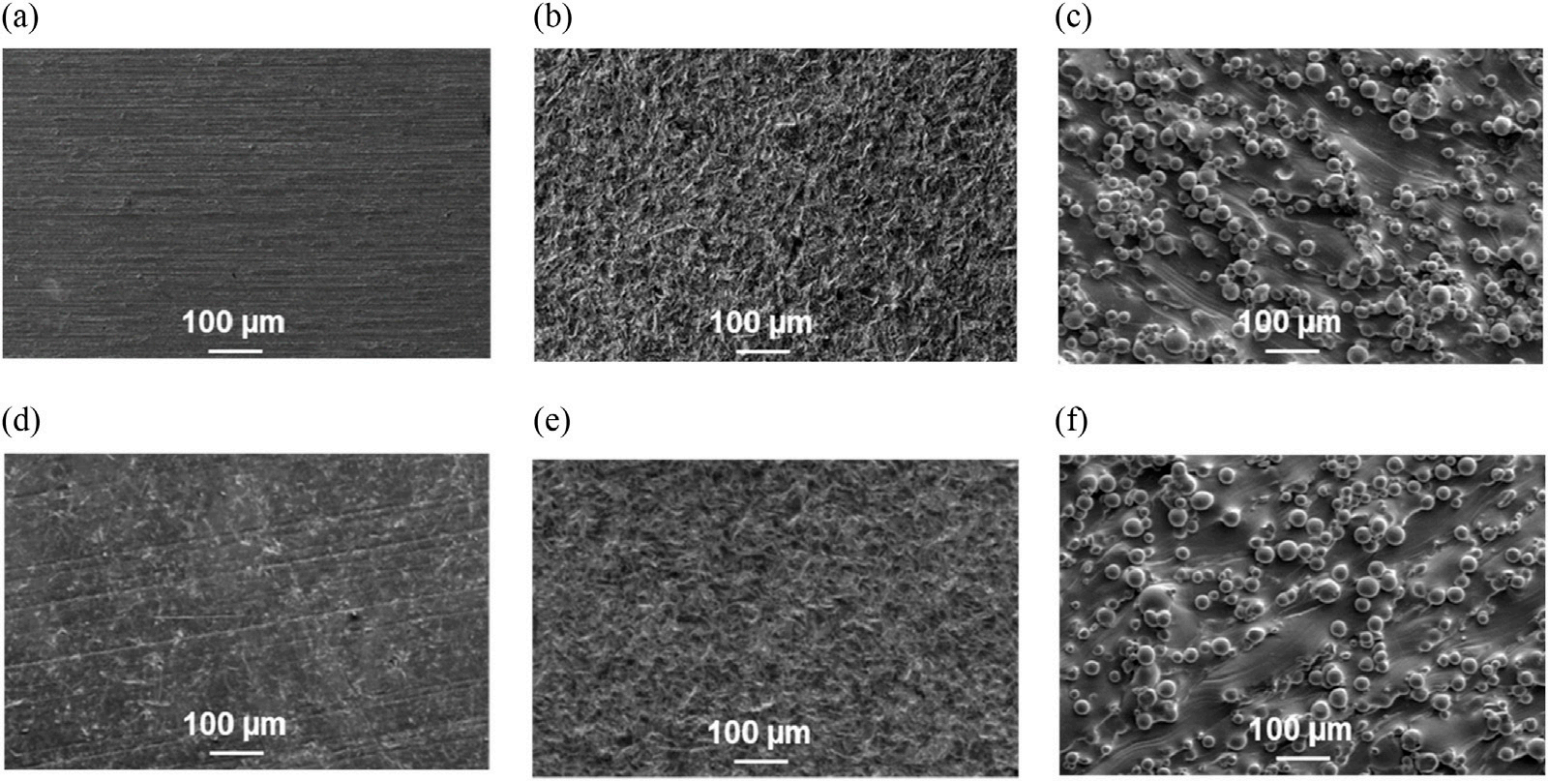
SEM images of **(a, d)** MTi surface **(b, e)** SBTi surface **(c, f)** AMTi15 surface; uncoated (upper row) and PEM coated (lower row).

SEM images in Figure 5 demonstrate significant differences in both macro- and microtopography of the Ti-6Al-4V discs substrates. Machined Ti-6Al-4V discs (Figure 5a) display an anisotropically grooved surface. On the contrary, both sandblasted (Figure 5b) and additively manufactured (Figure 5c) samples exhibit a highly rugged and anisotopically irregular surface.

#### 3.3.2 PEM-coated Ti-6Al-4V specimens

Polyelectrolyte multilayers (PEMs) are assembled through the layer-by-layer deposition of oppositely charged polyelectrolytes, offering a versatile platform for surface modification in biomedical applications. Their tunable physicochemical properties, including thickness, roughness, and mechanical stiffness, can be precisely controlled by adjusting parameters such as pH, ionic strength, and the specific polyelectrolytes used during assembly. This adaptability makes PEMs particularly suitable for tailoring surface characteristics to enhance cellular interactions and promote tissue integration. In the context of titanium-based implants, such as Ti-6Al-4V alloys, surface biofunctionalization is crucial to improve biocompatibility and encourage osseointegration. Among various PEM systems, those composed PAA and PAH have been extensively studied due to their ability to form stable multilayers with controllable properties. The assembly pH significantly influences the film’s characteristics; for instance, PEMs assembled at higher pH values tend to exhibit increased stiffness and promote better cell adhesion compared to those assembled at lower pH levels (Thompson et al., 2005). In tissue engineering, the PAA/PAH films have shown superior cell adhesion properties compared to other PEMs. When assembled at optimal pH levels, these multilayers promote excellent adhesion of fibroblasts and endothelial cells, making them suitable for biomedical applications (Thompson et al., 2005; Hajicharalambous et al., 2009). This enhanced cell compatibility is not universally observed in all PEM systems.

The (PAA/PAH)_5_ as applied on model Si-wafers is just a few nanometers thin (12.2 ± 2.1 nm) and mildly hydrophilic (static water contact angle 73 ± 2°, Table 3). Consistent with the low thickness of the coating, its average roughness is also minimal (1.7 ± 0.4 nm). AFM images in Supplementary Figure S2 demonstrate the homogeneous and nanostructured topography of the PEM coating. Both the PAH and PAA chains are fully ionized at pH 7.0, which ends up with a strong repulsion between the COO^−^ groups in the PAA chains and NH_3_^+^ groups in the PAH chains. This causes the polymer chains to adopt a fully extended conformation in solution, which is retained during surface adsorption, leading to extremely low thickness and roughness of the PAA/PAH films assembled at neutral pH. Applying nano-thin, nano-smooth PEM coating to MTi, SBTi, and AMTi substrates effectively preserves their inherent micromorphology (Figures 5d–f). Coating smooths surface contours, eliminates loosely bound titanium particles from AMTi materials, and enhances the surface chemical homogeneity. The PEM coating exhibits strong adhesion, conforming seamlessly to the Ti surfaces without defects such as scratches or delamination.

**TABLE 3:** Static water contact angles (CA, in degrees) of model Si-wafers and on Ti-6Al-4V discs with five different Sa values, uncoated and coated with (PAA/PAH)_5_ PEM coating.

Substrate	Contact angle of uncoated substrate	Contact angle of PEM-coated substrate
Si-wafer	64° ± 3°	73° ± 2°
MTi	73° ± 3°	45° ± 8°
SBTi	99° ± 4°	66° ± 5°
AMTi13	59° ± 5°	22° ± 4°
AMTi15	67° ± 5°	23° ± 3°
AMTi17	60° ± 7°	26° ± 5°

It is known that the surface roughness contributes to the material hydrophilicity, therefore we assessed the hydrophilicity of the uncoated MTi, SBTi and AMTi discs with different roughness, as well as the same discs coated with the PEM film by measuring the water contact angle (Table 3, representative contact angle images are presented in Supplementary Figure S3). All uncoated Ti-substrates are moderately hydrophilic, except SBTi, which is slightly hydrophobic, presumably due to modification of the surface chemical composition during sandblasting. Although ultra-thin, the PEM coating significantly enhances the hydrophilicity of all Ti-6Al-4V surfaces without altering their micro-roughness and morphology. The adhesion between a coating and an implant’s surface is paramount among mechanical properties, as it dictates the implant’s performance and functionality. If the coating detaches from the substrate - a phenomenon known as delamination - it can result in implantation failure and compromised long-term stability. To assess this adhesion strength, we employed the pull-off test, which quantifies the force per unit area necessary to separate the coating from its substrate. This method aligns with the ASTM F1147-05 standard, designed to evaluate the adhesion of coatings to solid metal substrates or the internal cohesion of a coating under tension perpendicular to the surface plane.

The tensile load applied to detach the uncoated AMTi cylinder from the control cylinder, also known as adhesive strength, was 18.8 ± 0.1 MPa. The adhesive strength of the PAA/PAH-coated AMTi-cylinder was 19.5 ± 0.2 MPa, higher than that of the uncoated cylinder. The adhesive strength of the uncoated MTi sample was 25.3 ± 2.1 MPa and remained unchanged after the application of PAA/PAH coatings (25.4 ± 2.5 MPa). According to ISO standards, hydroxyapatite coatings applied to solid metal substrates must exhibit a minimum adhesion strength of 15 MPa to be deemed suitable for implant applications. The PEM coating discussed herein demonstrate adhesion strengths that exceeds this requirement, positioning it as promising candidate for application on medical devices.

#### 3.3.3 Effect of roughness and PEM coating on the cell behavior

There is a number of publications on the effect of surface roughness on cell adhesion, proliferation, viability, migration, and differentiation (Celles and dos Reis, 2024; Lu et al., 2020; Metwally et al., 2020). Despite the large amount of data collected, they led to controversial conclusions that might originate from the various ranges of nano- and micro-roughness, topographies, materials, and cell types studied. Here we investigated, on the one hand, the effect of roughness of AMTi-materials at a narrower micro-scale (12 μm, 15 μm, and 17 µm), and on the other hand, the effect of roughness at a much broader nano-to micro-scale (0.5 µm, 2 μm and 15 µm), on cell adhesion and proliferation of different cell types. The effect of surface biofunctionalization with the PAA/PAH coating was also investigated.

##### 3.3.3.1 Micro-scale

A notable aspect of this study is the assessment of both the individual and combined impacts of varying microscale surface roughness (12 μm, 15 μm, and 17 µm) and the application of a nano-thick PAA/PAH coating on the cytotoxicity and cytocompatibility of additively manufactured titanium materials. This was evaluated by studying the viability of L929 fibroblasts, MC3T3 preosteoblasts, and human dental pulp stem cells (hDPSCs). hDPSCs are a relevant model for evaluating osteogenic potential and can differentiate into various cell lineages, including osteoblasts (Awais et al., 2020). The use of hDPSCs offers valuable insights into the interaction between biomaterials and cells which are important for bone regeneration and repair.

**FIGURE 6:**
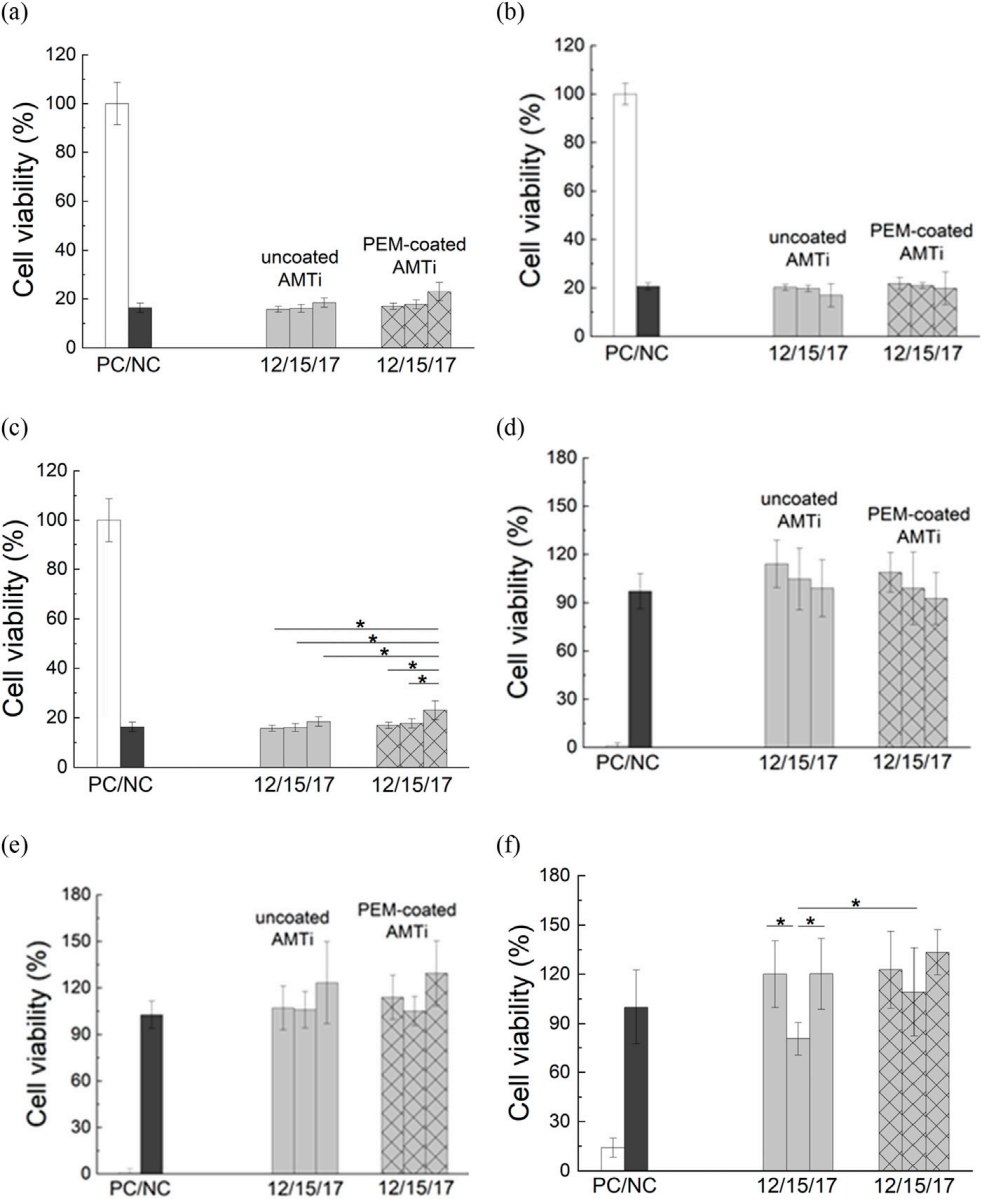
Cell viability of **(a, d)** L929 murine fibroblast **(b, e)** MC3T3 preosteoblasts; and **(c, f)** human dental pulp stem cells (hDPSCs) on toxic positive control (white columns), non-toxic negative control (dark grey columns), non-coated AMTi discs with roughness 12 μm, 15 μm and 17** **µm (light gray columns), and PAA/PAH-coated AMTi discs with roughness 12 μm, 15 μm and 17** **µm (light gray hatched columns), estimated by LDH assay **(a-c)** and XTT assay **(d-f)**, (* designates values that are significantly different, p < 0.05).

The cell cytotoxicity and viability of the 3 cell types studied on AMTi-materials were assessed by LDH and XTT assays. Both assays revealed that all cell types keep their viability on non-coated and PEM-coated AMTi-substrates equal to that on the non-toxic negative control (Figure 6) indicating a robust adherence on the specimens. Different roughness, as well as PAA/PAH coating, have neither an inhibitory effect on cell adhesion or viability nor a toxic effect on the cells.

Taken together, the results from the LDH assay indicate that the samples can be classified as biocompatible according to the cytotoxicity threshold defined in EN ISO 10993–5 (≥70% cell viability). The variation of the surface roughness of additively manufactured Ti-6Al-4V in the range of 12–17 µm has no statistically significant impact on the cytotoxicity of any of the 3 cell types investigated. The XTT assay shows that cell viability, proliferation, and metabolic activity is not significantly influenced either by the variation of surface micro-roughness not by the application of PAA/PAH coating.

Prior studies unite around the statement that osteoblastic cell adhesion, growth, and proliferation are positively correlated to surface roughness (Nöth et al., 1997). Rough surfaces encourage the entrapment of proteins and adhesion of osteogenic cells (Linez-Bataillon et al., 2002). However other studies report a reverse linear correlation between osteoblasts proliferation rate and surface roughness like the one (Boyan et al., 1999; Anselme, 2000). The discrepancies in the various studies may be attributable to differences in the examined cell species and the range of roughness. The results generated in this study refer to specimens with a high macro-topological level (*R*_a_ from 12 to 17 µm) therefore comparison with results described in the literature might not be relevant.

##### 3.3.3.2 Submicron-to-micron-scale

A straightforward technique that is frequently used to prepare the surface of dental implants is sandblasting accelerating osteoblast attachment, thus leading to an increase in osseointegration (Vogler, 1998). Here, we used sandblasting as a method to develop surfaces with an intermediate roughness between that of machined and additively manufactured Ti-6Al-4V (see Table 3).

**FIGURE 7:**
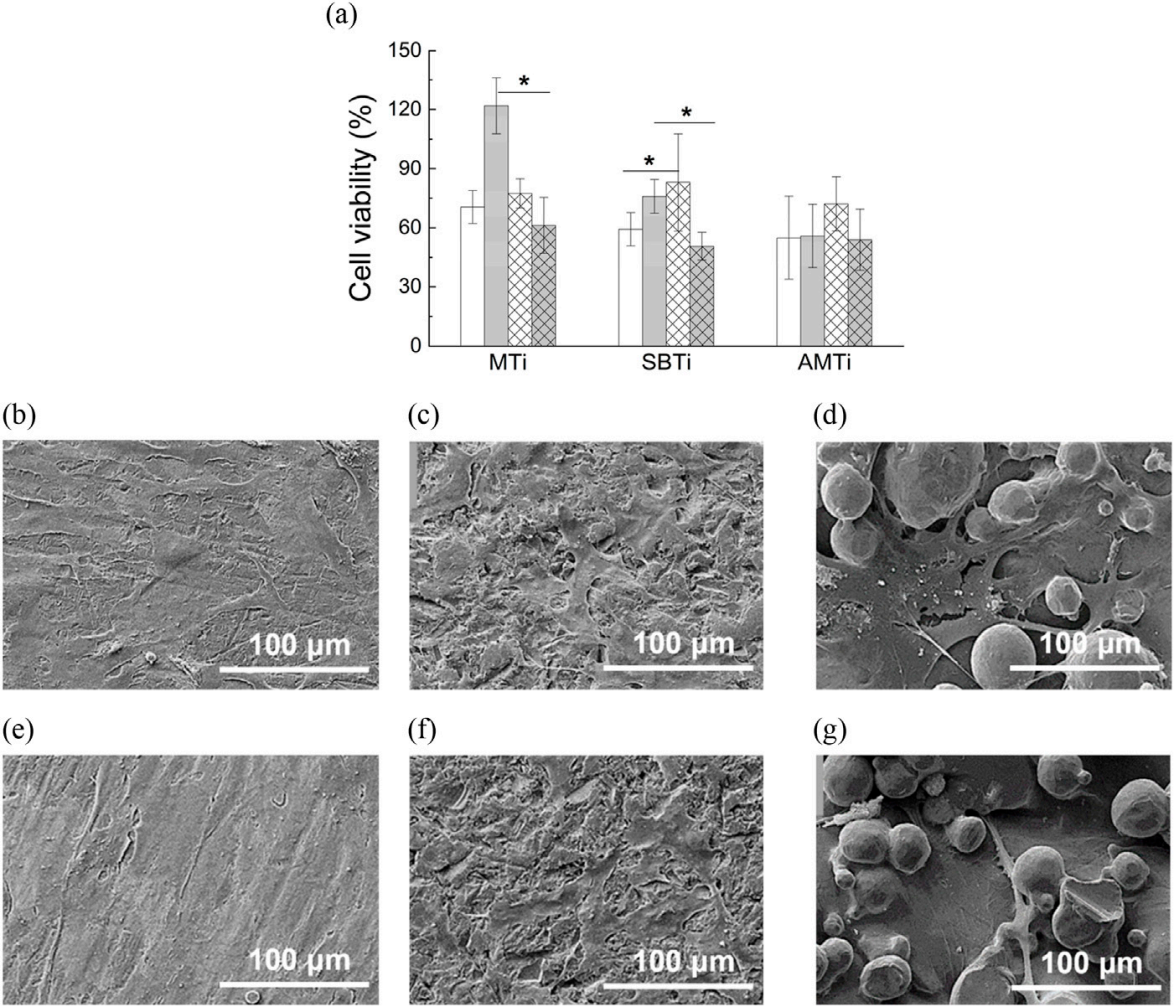
**(a)** NHOst viability (relative to the positive control) after 24 h of cultivation (white columns), showing the extent of cell adhesion and after 72 h of cultivation (gray columns), showing the extent of cell proliferation, examined on MTi, SBTi and AMTi discs, non-coated (normal bars) and coated with PEM coating (hatched bars) (* designates values that are significantly different, p < 0.05). SEM images of NHOst cultured for 24 h (upper row) and 72 h (bottom row) on Ti-6Al-4V discs with increasing roughness: **(b, e)** MTi, **(c, f)** SBTi, and **(d, g)** AMTi. Magnification 1,200×.

It is evident from Figure 7a) that NHOst adhesion was not affected by the significant increase in surface roughness for either the uncoated or PEM-coated Ti-6Al-4V specimen. It looks like the PEM-coating enhanced the initial cell attachment on the Ti-6Al-4V specimen but statistical analysis revealed that significant increase of osteoblast adhesion (by 40%) is observed only on sandblasted Ti-6Al-4V specimens relative to the uncoated SBTi. As for the proliferation, a dramatical suppression was found for the uncoated SBTi and AMTi compared to the smooth MTi samples. All PEM-coated samples demonstrated the same level of cell viability regardless the surface roughness.

The findings regarding the relationship between surface hydrophilicity and the behavior of osteoblasts are in agreement with the so-called Berg limit (Vogler et al., 1995), which was established in the context of protein adsorption and cell adhesion. This limit states that surfaces lose their ability to bind hydrophilic and hydrophobic proteins when their contact angle falls below 60°–65° (Vogler et al., 1995). The functionalization of MTi and AMTi biomaterials with the PAA/PAH coating lowered the contact angle much below this threshold, thus suppressing the strength of cell adhesion, and consequently the proliferation ability. Uncoated SBTi biomaterial appeared hydrophobic and numerous scientific researches reveal suppressed cell adhesion and spreading on hydrophobic surfaces (Barrias et al., 2009; Glenske et al., 2018). The adsorption of proteins that promote cell adhesion, such as fibronectin and vitronectin, is hampered by hydrophobic surfaces’ generally low surface energy (Barrias et al., 2009). PEM coating increases the hydrophilicity of the originally hydrophobic SBTi substrate, thereby enhancing NHOst adhesion.

Our results on the NHOst behavior on uncoated Ti-6Al-4V specimen with increasing submicron-to-micron roughness align well with the literature. Evidence is accumulating to suggest a favorable cellular response to submicron and nanoscale roughness compared to micron-scale roughness. Alveolar bone osteoblasts cultured on surface-treated zirconia polycrystals with varying submicron average roughness (from 0.3 to 0.8 µm) and morphology showed comparable initial attachment efficiency, regardless of the surface properties (Rabel et al., 2020). Through deep correlation analysis, it was found that proliferation is related not so much to surface roughness as to surface enlargement and texture, with lower surface expansion and surface roughness anisotropy supporting better cell proliferation (Rabel et al., 2020). A systematic decrease in the number of attached cells, as well as proliferation levels, has been reported for MG-63 human osteoblast-like cells grown on Ti-6Al-4V discs with increasing roughness (Martin et al., 1995).

In this study, the SBTi and especially the AMTi are characterized by relatively high surface enlargement and anisotropy of the surface structures, both contributing to the suppression of NHOst proliferation. Regarding the AMTi surfaces, we assume that lateral space limitations restrict the osteoblasts’ ability to adhere effectively and proliferate. As shown in Figures 7b–g), approximately 50% of the AMTi surface is covered with Ti-6Al-4V beads, leaving only the remaining 50% available for the cells.

SEM images reveal that NHOst cells are closely spread and fully flattened on the MTi surface, exhibiting numerous cytoplasmic extensions and filopodia that follow the direction of the grooves (Figures 7b, e). On the SBTi surfaces the cells spread less and in more isotropic directions, developing fewer cytoplasmic extensions compared to the MTi substrates (Figures 7c, f). In contrast, for AMTi, the lack of wide smooth areas causes osteoblasts to be suspended by their filopodia attached to the Ti-6Al-4V beads, resulting in weak adhesion and suppressed proliferation (Figures 7d, e). SEM images of NHOst cells on the same three surfaces functionalized with PAA/PAH coating are not shown because they appear similar to the images presented in Figures 7b–g).

**FIGURE 8:**
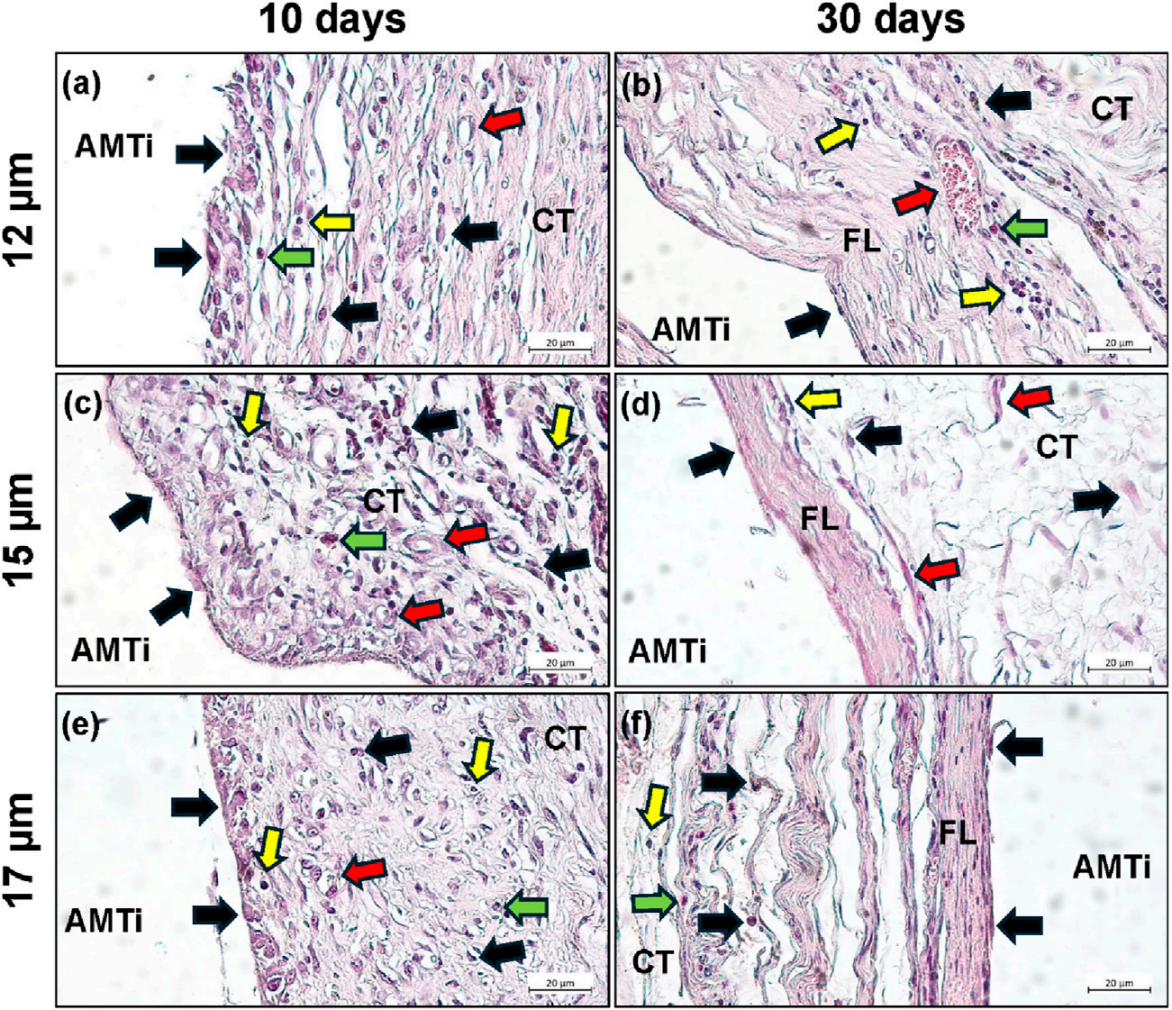
Representative histological microphotographs of the tissue reactions to the additive manufactured Ti-6Al-4V discs (uncoated- AMTi) at day 10 (left column) **(a,c,e)** and day 30 **(b,d,f)** post-implantation (right column) within the subcutaneous connective tissue (CT). Black arrows = macrophages, red arrows = blood vessels, green arrows = granulocytes, yellow arrows = lymphocytes (HE-stainings, magnification x400, scalebars = 20 µm).

Finally, the *in vivo* results showed that all the Ti-6Al-4V specimens induced comparable tissue reactions without any differences related to the roughnesses (Figure 8). At day 10 post-implantation, a moderate inflammatory tissue reaction pattern including mainly macrophages at the material-tissue-interfaces and within the surrounding connective tissue beside lower numbers of granulocytes and lymphocytes in concert with a mild vascularization was detectable in all study groups (Figure 8). This reaction pattern especially at this early time point has already been described and seems to be more related to the surgical procedure and the early wound healing processes around the implants. This is even more evident from the tissue reaction on day 30, which shows a flattening of the reaction or a slightly fibrotic tissue reaction with a reduced number of reactive cells in all study groups. However, this has already been shown in the case of other biomaterials and does not indicate either altered biocompatibility or reduced regenerative functionality (Vogler, 1998).

## 4 Discussion

### 4.1 Influence of mechanical properties on implant performance

The investigation of the mechanical properties and surface modifications of Ti-6Al-4V highlights the asymmetry between tensile and compressive behavior, which has been observed in various studies (Suresh et al., 2021). This asymmetry remains crucial and should be further considered in the development of medical implants, especially since the reduced Young’s modulus in the compressive direction could influence the long-term mechanical performance of implants subjected to repeated loading.

The α-β microstructure observed after heat treatment not only confirms previous findings but also emphasizes its role in improving the material’s mechanical properties, such as hardness. This enhancement is particularly important for applications where high strength and wear resistance are critical, such as load-bearing medical implants. The stability and hardness of this microstructure suggest its ability to withstand long-term mechanical stresses in clinical settings. However, future studies should focus on the effects of this microstructure on the long-term stability of implants under various clinical conditions.

The fatigue results confirm the reliability of Ti-6Al-4V for applications requiring long-term cyclic loading, such as in load-bearing implants. Within our research group, models for predicting material damage and understanding the tissue-implant interface are central to designing patient-specific implants. The fatigue properties obtained in this study serve as valuable experimental benchmarks for validating numerical simulations, providing essential insights for improving implant design, durability, and safety in medical applications.

### 4.2 Fatigue resistance and computational model efficiency

A particularly notable finding in this study is the high computational efficiency of the proposed model. To enhance computational efficiency, we implemented a time-to-cycle transformation, enabling the prediction of high-cycle fatigue behavior without the need for exhaustive cycle-by-cycle simulations. Quasi-static processes were assumed to simplify calculations, with the material model accounting for damage evolution, plastic strain, and hardening effects. Simulations were completed in just 18,024-time steps for a stress amplitude of 350 MPa, significantly reducing the number of time steps compared to the 1,254,380 required in conventional cycle-by-cycle approaches. The simulation results were rigorously validated against experimental S-N curves derived from high-cycle fatigue tests. The predicted fatigue life demonstrated a strong correlation with experimental data, with an error margin of less than 3%. This consistency underscores the reliability of the simulation framework for predicting fatigue behavior under realistic conditions. This efficiency, combined with consistent accuracy, highlights the model’s potential for predicting fatigue behavior under realistic loading conditions in additively manufactured materials. These findings support the application of the model to optimize implant designs, where long-term fatigue performance is critical, enabling precise, reliable predictions while reducing computational demands.

### 4.3 Surface hydrophilicity and roughness effects

The investigation of the surface hydrophilicity of PEM-coated MTi samples with low surface roughness shows that surface roughness significantly impacts the contact angle, with increasing Ra values enhancing the hydrophilicity of the samples. The sand-blasted Ti-6Al-4V samples are more hydrophobic than the machined samples. The additively manufactured Ti-6Al-4V samples, however, are significantly more hydrophilic than the machined and sand-blasted. According to the Wenzel model accounting for the contribution of the surface roughness component to the contact angle, increasing the roughness exaggerates the hydrophilicity of hydrophilic surfaces with the same chemical composition. Our data on the contact angle of non-coated as well as coated MTi and AMTi agree with the Wenzel rule, the impact of roughness being more pronounced on the PEM-coated samples due to their increased hydrophilicity compared to the non-coated samples (Table 3). However, the sandblasted samples behave somewhat differently and the reason can be found in the changed surface composition of these samples due to the traces of the blasted particles left on the treated surface (Guo et al., 2015). These findings highlight the importance of surface characteristics in modulating material hydrophilicity, a factor crucial for osseointegration in biomedical applications.

### 4.4 Cellular responses to surface roughness

Prior studies unite around the statement that osteoblastic cell adhesion, growth, and proliferation are positively correlated to surface roughness (Nöth et al., 1997). Rough surfaces encourage the entrapment of proteins and adhesion of osteogenic cells (Linez-Bataillon et al., 2002). However other studies report a reverse linear correlation between osteoblasts proliferation rate and surface roughness like the one (Boyan et al., 1999; Anselme, 2000). The discrepancies in the various studies may be attributable to differences in the examined cell species and the range of roughness. The results generated in this study refer to specimens with a high macro-topological level (*R*_a_ from 12 μm to 17 µm) therefore comparison with results described in the literature might not be relevant.

The study’s findings support the notion that surface roughness significantly influences cellular responses on Ti-6Al-4V implants, though effects vary depending on the roughness scale and surface treatment. While sandblasting enhanced initial osteoblast adhesion, it also decreased proliferation, a phenomenon consistent with findings that hydrophobic surfaces and high surface roughness reduce protein adsorption and cell spreading (Rabel et al., 2020; Martin et al., 1995). Furthermore, AMTis highly anisotropic and enlarged surface structures contributed to reduced proliferation, as limited lateral space restricted cell attachment.

Although nanoscale and submicron roughness are widely reported to promote osteogenic cell attachment, the high micro-scale roughness in this study resulted in diminished cell proliferation, highlighting the need for precise control of surface topography.

### 4.5 Challenges and limitations

Precise manufacturing of patient-specific implants requires advanced technologies such as additive manufacturing, but they are prone to defects and process variability. From a biological perspective, the long-term stability and integration of an implant mostly depend on the biological compatibility and the custom-fit design to match the individual patient’s anatomical requirements. Additionally, the customized implants’ design and manufacturing process is time-consuming, which can be particularly problematic in urgent clinical situations. Nevertheless, the production and development of personalized implants are currently cost-intensive and difficult to scale up. For the authorization process of patient-specific implants, strict regulatory requirements include extensive testing and verification.

### 4.6 Implications and future directions

Overall, this study highlights how both, the mechanical and surface properties of Ti-6Al-4V implants contribute to their performance in biomedical applications. Mechanical strength, fatigue resistance, and computational efficiency are critical for developing reliable load-bearing implants. Surface characteristics, especially roughness and hydrophilicity, directly influence cell adhesion and proliferation, impacting osseointegration. Future research should continue to investigate these properties to enhance implant safety, durability, and biocompatibility, with an emphasis on refining surface topography for optimal biological response.

## 5 Conclusions and outlook

This study demonstrated that powder bed fusion laser-based process (PBF-LB/M) Ti-6Al-4V is a viable material for dental and maxillofacial implants, exhibiting strong mechanical performance and excellent fatigue resistance. Quasi-static tests revealed a Young’s modulus of approximately 110 GPa, and tensile strength of 870 MPa, with superior compressive strength of 1,250 MPa. Fatigue life modeling and experimental validation confirmed the material’s long-term stability under cyclic loading. Surface modifications like sandblasting, enhanced cell viability and osseointegration by reducing the roughness values of 12.5 µm–17.4 µm, as observed in in-vitro studies. These findings support the use of Ti-6Al-4V for custom-made implants, ensuring both mechanical reliability and enhanced biological integration for improved patient outcomes.

Applying advanced modeling techniques could further refine predictions of implant longevity, enhancing the reliability and patient specificity of solutions for dental and maxillofacial applications. Future studies should also focus on assessing long-term mechanical stability under physiological loading conditions to ensure optimal clinical safety and effectiveness of patient-specific implants.

## Data Availability

The original contributions presented in the study are publicly available. This data can be found here: https://doi.org/10.25835/6nqow7m4.
