# Supplementary material for: Characterization and modeling of additively manufactured Ti-6Al-4V alloy with modified surfaces for medical applications
Source: Front Bioeng Biotechnol. 2025 Apr 7;13:1526873. doi: 10.3389/fbioe.2025.1526873 (PMC12010145; doi:10.3389/fbioe.2025.1526873)
Supplement: Supplementary file 1 [file DataSheet1.docx]

Supplementary Material

| Stress amplitude in experiment [MPa] | Displacement load in simulation [mm] |
| --- | --- |
| 350 | 0.13308 |
| 400 | 0.15210 |
| 450 | 0.17111 |
| 550 | 0.20912 |
| 600 | 0.22814 |
| 650 | 0.24716 |

**Table S.1:** Load amplitudes for the simulation.

| a) | b) | c) |
| --- | --- | --- |
| 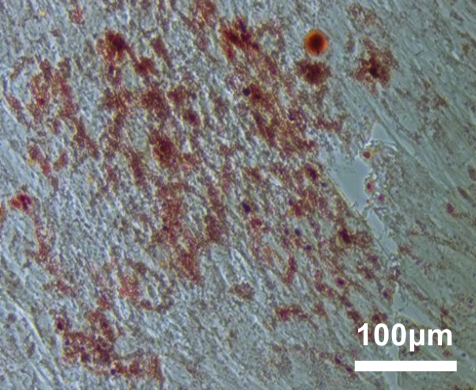 | 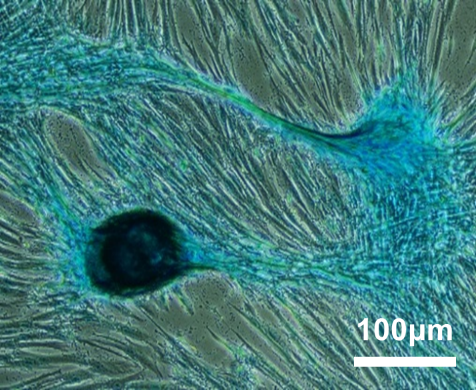 | 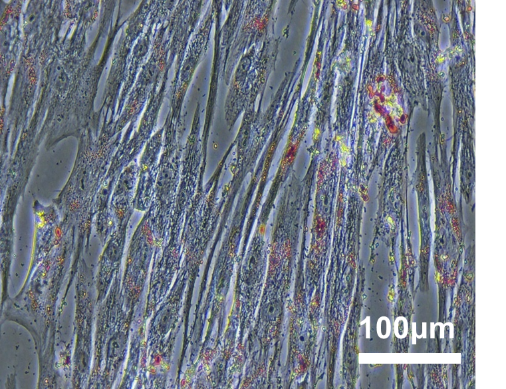 |
| **Figure S.1:** a) Osteogenic; b) chondrogenic; c) adipogenic differentiation of human dental pulp cells (hDPC). | | |

| a) | b) |
| --- | --- |
| 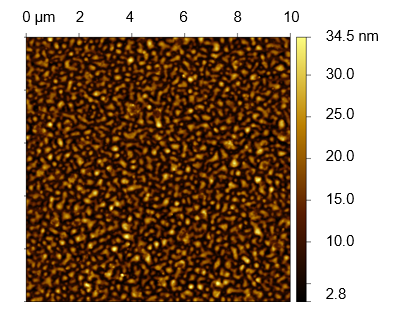 | 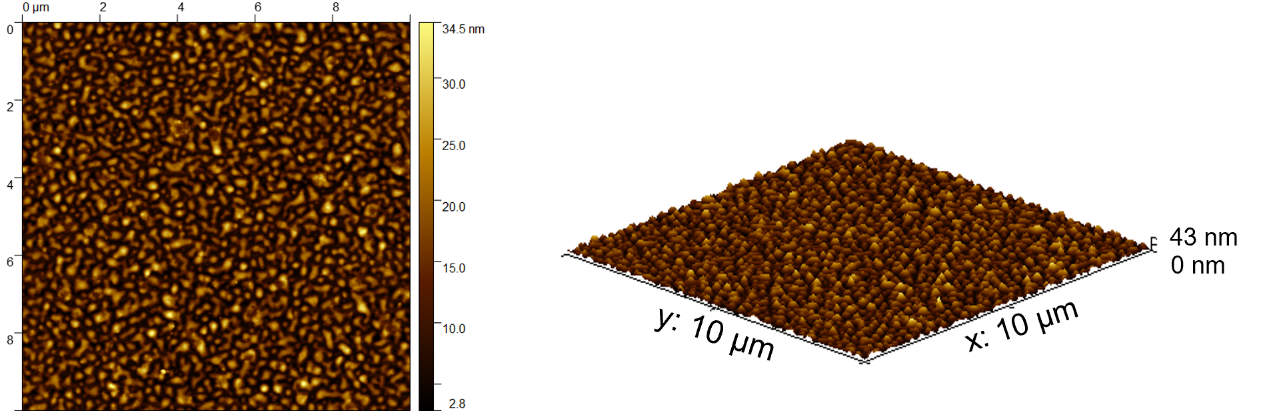 |

**Figure S.2:** a) AFM height image and b) AFM 3D topography image of (PAA/PAH)_5_ coating on Si-wafer.

| Substrate | Water droplet on  uncoated substrate | Water droplet on  PEM-coated substrate |
| --- | --- | --- |
| **Si-wafer** | 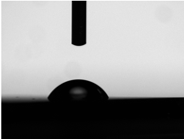 | 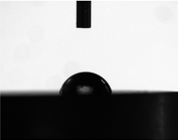 |
| **MTi** | 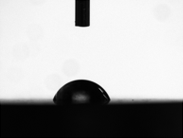 | 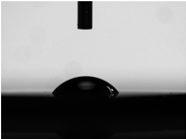 |
| **SBTi** | 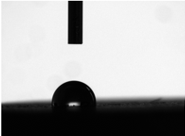 | 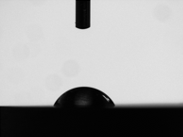 |
| **AMTi13** | 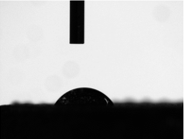 | 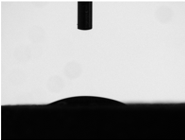 |
| **AMTi15** | 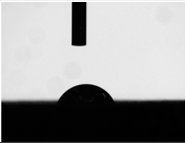 | 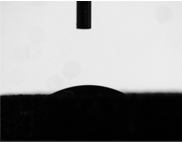 |
| **AMTi17** | 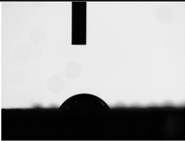 | 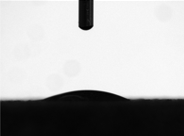 |

**Figure S.3:** Representative images of static water droplets on model Si-wafers and on Ti-6Al-4V discs with five different S_a_ values, uncoated and coated with (PAA/PAH)_5_ coating.
